# Preterm Piglets Born by Cesarean Section as a Suitable Animal Model for the Study of Iron Metabolism in Premature Infants

**DOI:** 10.3390/ijms252011215

**Published:** 2024-10-18

**Authors:** Xiuying Wang, Małgorzata Lenartowicz, Rafał Mazgaj, Magdalena Ogłuszka, Dominika Szkopek, Kamil Zaworski, Zuzanna Kopeć, Beata Żelazowska, Paweł Lipiński, Jarosław Woliński, Rafał Radosław Starzyński

**Affiliations:** 1 Laboratory of Iron Molecular Biology, Department of Molecular Biology, Institute of Genetics and Animal Biotechnology, Polish Academy of Sciences, 05-552 Jastrzębiec, Poland; x.wang@igbzpan.pl (X.W.); r.mazgaj@igbzpan.pl (R.M.); z.kopec@igbzpan.pl (Z.K.); b.zelazowska@igbzpan.pl (B.Ż.); p.lipinski@igbzpan.pl (P.L.); 2 Laboratory of Genetics and Evolutionism, Institute of Zoology and Biomedical Research, Jagiellonian University, 30-387 Kraków, Poland; 3 Department of Genomics and Biodiversity, Institute of Genetics and Animal Biotechnology, Polish Academy of Sciences, 05-552 Jastrzębiec, Poland; m.ogluszka@igbzpan.pl; 4 Laboratory of Large Animal Models, The Kielanowski Institute of Animal Physiology and Nutrition, Polish Academy of Sciences, 05-110 Jabłonna, Poland; d.szkopek@ifzz.pl (D.S.); j.wolinski@ifzz.pl (J.W.); 5 Department of Animal Physiology, The Kielanowski Institute of Animal Physiology and Nutrition, Polish Academy of Sciences, 05-110 Jabłonna, Poland; k.zaworski@ifzz.pl

**Keywords:** piglet, cesarean section, animal model, iron, preterm

## Abstract

Preterm infants are most at risk of iron deficiency. However, our knowledge of the regulation of iron homeostasis in preterm infants is poor. The main goal of our research was to develop and validate an animal model of human prematurity to assess iron status in preterm infants. We performed a cesarean section on sows on the 109th day of pregnancy, which corresponds to the last trimester of human pregnancy. Preterm piglets showed decreased body weight, red blood cell indices, plasma iron level and transferrin saturation. Interestingly, higher hepatic and splenic non-heme iron content and plasma and hepatic ferritin levels were found in premature piglets compared with term ones. In addition, premature piglets showed higher mRNA levels of iron-regulatory hormone hepcidin in the liver than term animals, which have not been reflected in higher plasma hepcidin-25 levels. We also showed changes in hepcidin regulators, including hepatic bone morphogenetic protein 6, plasma erythroferrone and growth differentiation factor 15 in preterm piglets. Consequently, no difference was observed in iron-exporter ferroportin levels in the spleen and liver. Overall, it seems that premature piglets show a pattern of iron metabolism characteristic of functional iron deficiency and iron accumulation in the tissue.

## 1. Introduction

Due to its redox properties, iron is an essential micronutrient involved in many key biochemical processes and cellular functions. Iron deficiency (ID) is one of the most widespread nutritional deficiencies and affects all age groups. Children up to 5 years of age are the most affected due to high iron requirements in this period of rapid growth. Perinatal perturbations in iron homeostasis may result in alterations in cognitive functions and neurodevelopment [1]. Preterm infants are prone to develop ID anemia in the first 4 months of life due to lower iron stores at birth compared with term infants because most fetal iron stores are built up as a result of iron transfer from the mother during the third trimester of gestation. This transfer may be interrupted by gestation shortening. During development, ID negatively affects the growth and function of many organs, including the liver, heart and brain [2].

Designing strategies for iron supplementation in neonates is complicated. Detailed investigations of this issue during the neonatal period after normal and shortened pregnancies, as well as the search for suitable animal models for neonatal iron supplementation studies, are urgently required. A proper animal model of human preterm physiology should include viable neonates similar to preterm human infants and have a body size that allows comparative monitoring, blood sampling and clinical interventions. Large animals such as rhesus monkeys [3], sheep [4] and dogs [5] have been used for modeling human physiology and nutrition. However, the availability of these animals is very restricted, and their cost is very high, thus limiting the feasibility of experiments using these models. In contrast, pigs are available and relatively cheap to maintain animals, and their usefulness in studies on human prematurity has been proven by Eiby et al. [6]. Piglets provide a clinically relevant model of preterm neonatal physiology where the maturation of multiple organ systems is similar to human early preterm infants [6]. In particular, although the human placenta is hemochorial and the pig placenta is epitheliochorial, the key proteins involved in iron transport function similarly across species despite these structural differences [7]. The results of several studies [8,9], including our own [10,11,12,13], indicate that term newborn piglets are a suitable model with which to explore iron metabolism in the neonatal period. Here, we attempt to validate piglets born by cesarean section as a pig model of prematurity, reflecting the mechanisms of ID in human premature infants as much as possible. Moreover, the piglets obtained by cesarean section and kept in incubators can be used in follow-up studies during the neonatal period.

## 2. Results

### 2.1. Substantial Decrease in Body Weight in Premature Piglets

**Figure 1:**
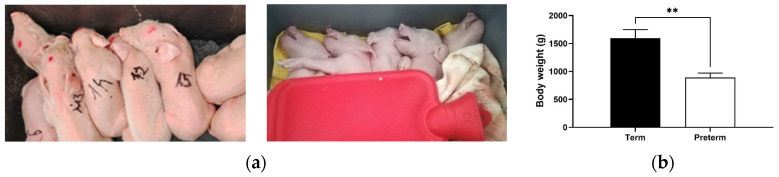
Term (left) and preterm (right) piglets appearance (**a**) and their body weight (**b**). (**a**) The photos were taken just after birth. Premature piglets need heating pads to maintain body temperature. (**b**) Data are presented as the mean ± SEM (*n* = 6). ** asterisk denotes a statistically significant difference at *p* < 0.01 between term and preterm piglets.

Premature piglets were delivered at 95% gestation (109 d of 115.4 d gestation) by cesarean section to mimic human late preterm birth. Compared with full-term delivery, premature birth by cesarean section led to a drop in body weight by 44% (*p* < 0.01; Figure 1). Premature piglets presented spontaneous respiration upon delivery and were hemodynamically stable.

### 2.2. Changes in Red Blood Cell (RBC) and Reticulocyte Indices in Premature Piglets

**Figure 2:**
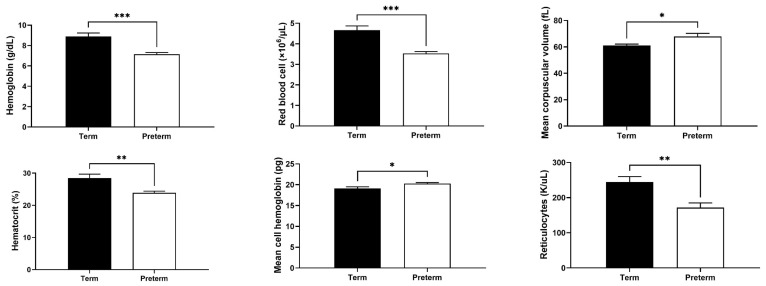
Red blood cell and reticulocyte indices. Data are presented as the mean ± SEM (*n* = 6). *, ** or *** asterisks denote statistically significant differences at *p* < 0.05, *p* < 0.01 or *p* < 0.001, respectively.

Hemoglobin level, RBC count, hematocrit value and reticulocyte count in preterm piglets were significantly reduced compared with term animals by 20, 24, 16 and 30%, respectively (*p* < 0.01; Figure 2). On the other hand, mean corpuscular volume (MCV) and mean cell hemoglobin values were higher in preterm piglets compared with full-term piglets by 11 and 6%, respectively (*p* < 0.05; Figure 2). Overall, RBC indices indicate the occurrence of anemia in preterm piglets.

### 2.3. Strong Decrease in Biochemical Plasma Iron Parameters in Premature Piglets

**Figure 3:**
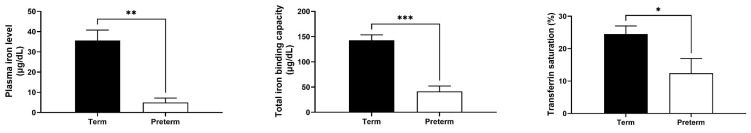
Blood plasma biochemical iron parameters. Data are presented as the mean ± SEM (*n* = 6). *, ** or *** asterisks denote statistically significant differences at *p* < 0.05, *p* < 0.01, or *p* < 0.001, respectively.

Premature piglets showed dramatically lower plasma iron levels (reduction by 86%), total iron binding capacity (TIBC) (reduction by 71%) and transferrin saturation (TSAT) values (reduction by 49%) than full-term piglets (*p* < 0.05; Figure 3). These results attest to a severe ID in preterm piglets.

### 2.4. Increased Hepatic Iron Status in Premature Piglets

**Figure 4:**
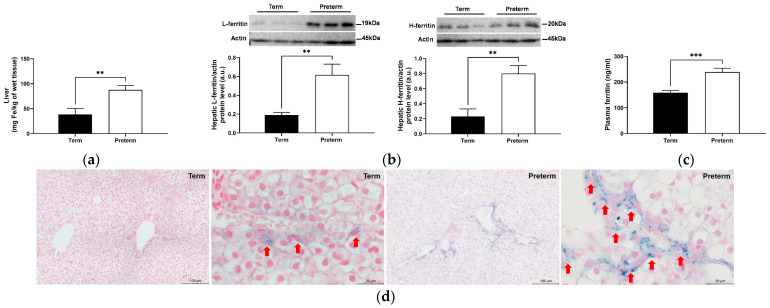
Hepatic non-heme iron content (**a**) and ferritin protein level (**b**), plasma ferritin level (**c**), and hepatic histological examination of iron loading (**d**). Data are presented as the mean ± SEM (*n* = 6). ** or *** asterisks denote statistically significant differences at *p* < 0.01 or *p* < 0.001, respectively. (**b**) Representative Western blot images and relative densitometric bar graphs of hepatic ferritin. Actin was used as a protein loading control. a.u., arbitrary units; H-ferritin, ferritin heavy chain; L-ferritin, ferritin light chain. (**d**) Non-heme iron deposits were detected by staining with Perls’ staining (blue stain) (original magnification: 200×. Scale bars = 100 μm). Iron deposits at high magnification are shown by red arrowheads (original magnification: 1000×; scale bars = 20 μm).

In preterm piglets, we noted that hepatic non-heme iron content was twice as high as that in term piglets (*p* < 0.01; Figure 4a). Hepatic ferritin level reflects iron abundance in the liver [14]. The assessment of protein levels of ferritin light chain (L-ferritin) and ferritin heavy chain (H-ferritin) in piglets’ livers by Western blotting largely confirmed the results of direct measurement of iron content. Preterm piglets showed substantial increases in levels of L- and H-ferritin compared with term animals (*p* < 0.05; Figure 4b). Blood plasma ferritin concentration, an indirect indicator of hepatic iron stores, was also elevated by 50% (*p* < 0.001; Figure 4c). Finally, microscopic analysis of liver sections stained for non-heme iron with Perls’ staining shows strong iron deposits in preterm piglets, whereas, in term animals, weaker staining of non-heme was found (Figure 4d).

### 2.5. Splenic and Bone Marrow Iron Status in Preterm and Term Piglets

**Figure 5:**
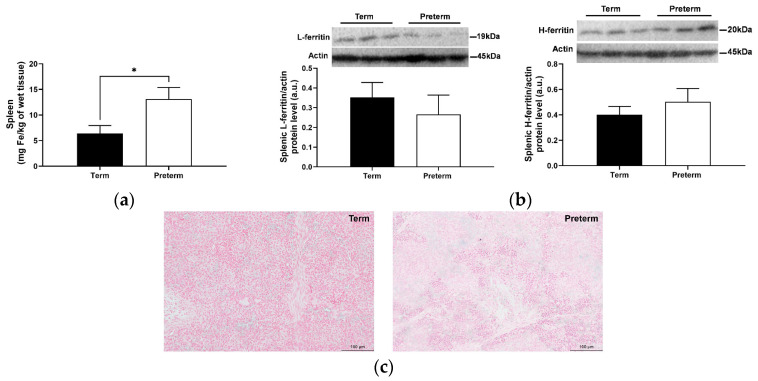
Splenic non-heme iron content (**a**), ferritin protein level (**b**), and histological examination of iron loading (**c**). Data are presented as the mean ± SEM (*n* = 6). * asterisk denotes a statistically significant difference at *p* < 0.05 between term and preterm piglets. (**b**) Representative Western blot images and relative densitometric bar graphs of splenic ferritin. Actin was used as a protein loading control. a.u., arbitrary units; H-ferritin, ferritin heavy chain; L-ferritin, ferritin light chain. (**c**) Non-heme iron deposits were detected by staining with Perls’ staining (original magnification: 200×. Scale bars = 100 μm).

Similar to the liver, a significant increase in non-heme iron content in the spleen was observed in preterm piglets (*p* < 0.05; Figure 5a). However, no increase in L- and H-ferritin protein levels was detected in these piglets compared with term animals (Figure 5b). Splenic non-heme iron was not detected by Perls’ staining either in preterm or in term piglets (Figure 5c). Similarly to the spleen, no iron deposits were detected in the bone marrow of either preterm or term piglets (Figure S1).

### 2.6. Changes in Levels of Hepcidin and Its Regulators in Premature Piglets

**Figure 6:**
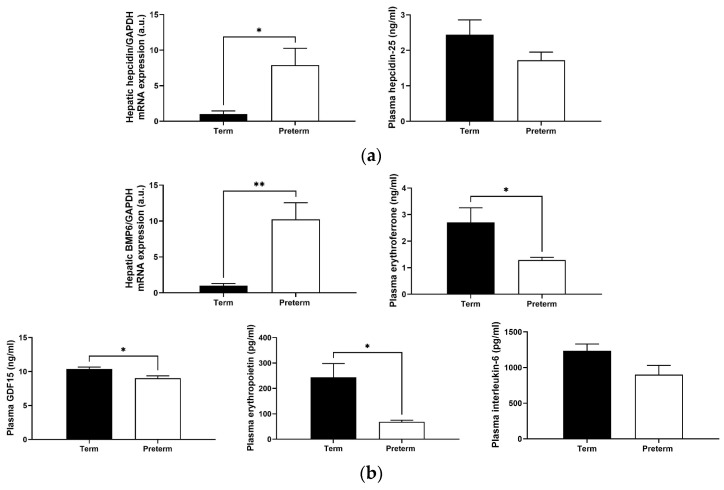
Hepcidin level (**a**) and its regulators (**b**). Data are presented as the mean ± SEM (*n* = 6). * or ** asterisks denote statistically significant differences at *p* < 0.05 or *p* < 0.01, respectively. a.u., arbitrary units; BMP6, bone morphogenetic protein 6; GDF15, growth differentiation factor 15.

Hepcidin, a peptide produced mainly by the liver in response to high iron content, is considered the main regulator of systemic iron homeostasis [15]. Hepatic hepcidin mRNA expression in premature piglets was nearly eight times that of term ones (*p* < 0.05), but no difference was found in the blood plasma hepcidin-25 concentration (Figure 6a). Relative to term piglets, the expression level of mRNA encoding for bone morphogenetic protein 6 (BMP6), a key endogenous inducer of hepcidin synthesis [16] in premature animals, was also significantly higher (*p* < 0.01; Figure 6b). In parallel, we found in the blood plasma decreased levels of erythroid hepcidin suppressors, such as erythroferrone (reduction by 52%) and growth differentiation factor 15 (GDF15) (reduction by 13%) (*p* < 0.05; Figure 6b). Furthermore, assessment of erythropoietin blood plasma concentration in preterm piglets revealed a substantial decrease compared with term ones (*p* < 0.05; Figure 6b). Finally, preterm and term piglets showed no difference in the levels of interleukin-6 and tumor necrosis factor α (Figure 6b and Figure S2), the inflammatory cytokines responsible for the induction of hepcidin during inflammation [17].

### 2.7. Similar Hepatic and Splenic Ferroportin Levels in Term and Preterm Piglets

**Figure 7:**
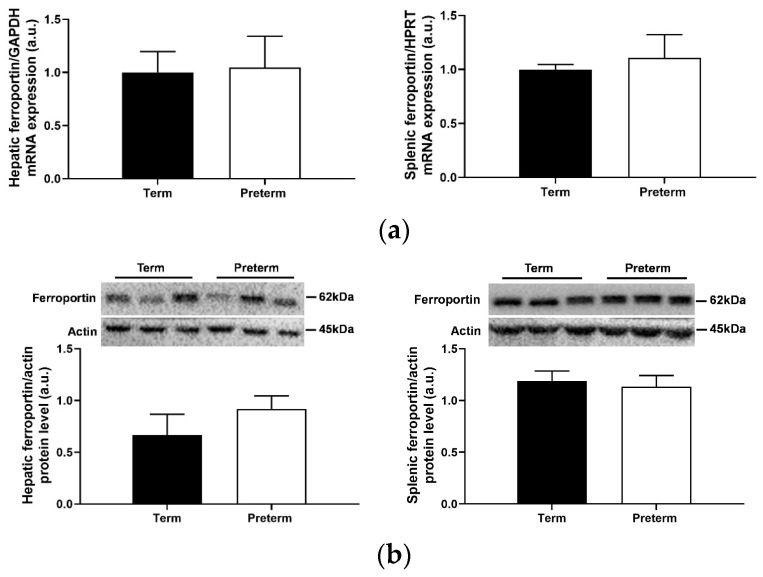
Ferroportin mRNA expression (**a**) and protein level (**b**) in the liver and spleen. Data are presented as the mean ± SEM (*n* = 6). (**b**) Representative Western blot images and relative densitometric bar graphs of hepatic and splenic ferroportin. Actin was used as a protein loading control. a.u., arbitrary units.

Ferroportin, the only iron exporter known in mammalian cells that transfers iron to plasma apo-transferrin, is a molecular target of hepcidin, which binds to this protein to induce its degradation, thus inhibiting iron release from exporting cells [18]. Analysis of both ferroportin mRNA and protein levels in the liver and spleen showed no difference between preterm and term piglets (Figure 7), which is consistent with similar blood plasma hepcidin levels in piglets from both investigated groups.

## 3. Discussion

The cesarean section is a common way to harvest preterm piglets. The first attempts were made by Eiby et al. [6], who developed a porcine model of preterm infants delivered by cesarean section at six gestational ages from day 91 to day 113. This experiment focused on anthropometric studies and showed that the degree of organ development and maturation is proportional to advanced age [6]. However, the regulation of iron metabolism in preterm piglets was not considered. In our case, piglets born by cesarean section on the 109th day of gestation started spontaneous breathing and did not show signs of hypoxemia or hemodynamic instability. It is worth noting that the shortening of pregnancy by an average of 6% translated into a significant decrease in the birth weight of premature piglets by 44% in our studies. Similarly, in humans, weight loss is observed depending on the degree of prematurity [19,20].

In order to validate our pig model of ID in prematurity, we first analyzed the RBC status in the blood plasma. We found that RBC count, hemoglobin level and hematocrit value were significantly lower in preterm piglets compared with full-term piglets. Similar results in piglets born by cesarean section after pregnancy lasting 111–112 days were reported by other authors [21]. Regarding human preterm infants, a similar hematologic pattern was reported in umbilical cord blood [22]. Our results also showed higher MCV and mean cell hemoglobin values in preterm piglets than in term animals. High MCV is characteristic of fetal erythrocytes and decreases with the duration of pregnancy [23]. As it was demonstrated by Alur et al. [20], hemoglobin and hematocrit values decreased, and MCV increased with the degree of human prematurity, which was in accordance with our results obtained in the piglet model of prematurity. Assuming that our pig model reflects human late prematurity, the analysis of umbilical cord blood of late preterm infants in the study of Rolim et al. [24] aligns perfectly with our results in preterm piglets.

Biochemical analysis of iron in the blood plasma or serum is important for the evaluation of both ID and anemia. Our results show that both plasma iron level and TIBC value were down-regulated in preterm piglets relative to term animals, consistent with results from studies on human preterm infants [25,26]. In both studies, a statistically significant increase in blood iron concentration and TIBC was documented with the advancement of pregnancy. In addition, lower TSAT was shown in preterm piglets compared to term ones in our study. ID is accompanied by low TSAT [27,28]. It has been suggested that TSAT values of <17% can attest to ID [29].

Examination of the hepatic and splenic iron content usually provides important information about iron status in the organism. Here, we have undertaken a comprehensive analysis of iron status in the liver of piglets, and surprisingly, we found that it is definitely higher in preterm piglets. In line with our results, data obtained by Georgieff et al. [30] indicated that the content of non-heme iron in the liver is higher in premature infants compared with term ones. Contrary to our results, Siddappa et al. [31] reported that in humans, umbilical cord serum ferritin concentration (an indirect indicator of hepatic iron stores) increases with gestational age, from an average of 63 to 171 μg/L at week 23 and week 41 of gestation, respectively. Similar to the liver, we found higher iron levels in the spleen of preterm piglets compared to term animals. However, this difference did not translate into decreased protein levels of both ferritin subunits. It is worth noting that splenic non-heme iron content is much lower than hepatic iron in either preterm or term piglets. Therefore, we suggest that changes occurring at a low iron level in the spleen are not reflected in fluctuations of ferritin protein level. We were unable to show changes in the level of non-heme iron in bone marrow smears, probably due to the very low level of iron in erythroid cells of the bone marrow. Similar results were obtained in preterm infants using Turnbull staining immediately after birth, clearly indicating low levels of non-heme iron in the bone marrow [32].

The lower non-heme iron content in term piglets compared to preterm piglets is somewhat puzzling. A possible explanation for this phenomenon could be an intensive mobilization of iron from hepatic iron stores to satisfy iron needs for increased production of RBCs (driven by increased erythropoietin levels, as shown in our study) at the end of pregnancy. Indeed, during mammalian prenatal development, definitive erythropoiesis occurs mainly in the fetal liver [33]. The liver is the main extramedullary erythropoietic tissue of the human fetus at midterm and continues to produce blood cells through the first week of life [34,35]. Furthermore, our data showing a considerable difference in the body weight between preterm and term piglets (about 700 g) strongly indicate that during the last 6 days of pregnancy, iron is intensively taken up from the liver to meet the demand for this microelement from other tissues. In summary, we propose that decreased non-heme iron levels in the liver in term piglets are due to exhaustive iron utilization in erythropoiesis accompanied by the shift of this iron fraction to the heme compartment in erythroid cells and intensive growth of fetuses during the very last period of pregnancy.

The next step in our study was to measure the expression of hepcidin, a small peptide hormone produced mainly by hepatocytes that orchestrate body iron fluxes by adjusting iron supply to body iron requirements [15,36]. Hepcidin binds to ferroportin to induce its degradation, thus inhibiting iron release from exporting cells [18]. We showed a statistically significant upregulation of hepcidin mRNA level in preterm versus term piglets in the liver. Considering that expression of hepcidin is transcriptionally controlled by elevated liver iron [37], this observation is consistent with high hepatic iron content in preterm animals.

It is known that BMP6 is the main transcriptional activator of hepcidin, which functions as an “iron sensor” whose transcription in liver sinusoidal endothelial cells is induced by high iron levels [16]. Here, we found that BMP6 is strongly induced in the liver of preterm piglets, which may explain the high mRNA expression of hepcidin in these animals. Contrary to iron, hepcidin expression can be suppressed by secreted erythroid factors, such as erythroferrone [38] and GDF15 [39]. We examined plasma levels of these factors and found their decline in preterm piglets. This implies that high hepcidin levels in these animals may also result from the attenuation of erythroid hepcidin suppressors. Kautz and Nemeth [38] described how erythroferrone synthesis is promoted by the release of erythropoietin, which then contributes to expanded erythropoiesis. Accordingly, blood plasma erythropoietin level was much lower in preterm piglets than in term animals, likely explaining low erythroferrone expression in the former. It is also worth noting that anemia of prematurity is characterized by reduced erythropoietin production [40,41]. A low plasma erythropoietin level is an important reason that nadir hematocrit values of preterm infants are lower than those of term infants [41], which is consistent with our results. In addition, some authors show that fetal serum hepcidin is likely triggered by the inflammatory effect of labor and delivery and that the level of hepcidin in preterm infants after cesarean section may be higher due to inflammation [17]. Apparently, this is not the case in our study, as the levels of inflammatory cytokines were similar in preterm and term piglets.

To our surprise, the measurement of bioactive hepcidin-25 concentration in piglet blood plasma showed very low levels of this peptide, with no statistically significant difference between animals from both experimental groups. In humans, fetal hepcidin levels in cord blood from preterm births were comparable to that of full-term births, suggesting a rather low level of hepcidin in the fetal stage [42]. Serum hepcidin values were also comparable and very low in both preterm infants with ID and preterm infants without ID [43]. In another of our studies, we also found that RNA levels of hepatic hepcidin increased, while blood hepcidin-25 levels did not change in 28-day-old piglets [44]. It should be emphasized that hepcidin is synthesized in the liver as an 84-aa pre-pro-hormone containing a typical N-terminal 24 amino acid endoplasmic reticulum targeting signal sequence and maturated by proteolysis through a consensus furin cleavage site (which is conserved in mammals) [45] to generate the bioactive 25-aa peptide secreted in the circulation [46]. We hypothesize that in the perinatal period, the control of hepcidin processing and release may be impaired, and that is why circulating hepcidin levels do not reflect its hepatic mRNA expression. Altogether, the analysis of blood plasma hepcidin in preterm and term piglets strongly suggests that this parameter may not be useful in the assessment of the perinatal systemic iron status of pigs.

As mentioned above, hepcidin achieves its function by binding to its cell membrane receptor, ferroportin, the sole iron exporter, which results in the endocytosis and degradation of ferroportin and consequently reduces iron release from macrophages, hepatocytes and enterocytes [18]. Here, we showed unchanged levels of ferroportin both in the liver and spleen of term and preterm piglets. This result is consistent with the stable concentration of hepcidin-25 in the blood plasma of piglets from both experimental groups. In the study by Tabbah et al. [47], ferroportin was localized postmortem in the liver of premature and full-term human neonates. The authors showed that hepatic ferroportin staining in term newborns was more prominent around the portal veins, while lower levels were observed around the central vein. In contrast, in the livers of newborns that died from early-onset neonatal sepsis, the ferroportin signal shifted to a diffuse pattern around the central vein [47].

Our study is the first attempt to characterize an animal model of prematurity based on the Polish Landrace breed. The data from the present study suggest that the validated pig model of prematurity largely mirrors systemic iron metabolism in preterm human infants. This model allows for in-depth biochemical and molecular analysis of tissue samples that are understandably impossible to obtain from humans. Obviously, we are aware of the limitations of our model, namely an extremely rapid growth rate of pig fetuses, which is not observed in human newborns. Furthermore, despite many similarities between pigs and humans, there are also some inconsistencies between pig and human organs. However, we believe that these differences do not undermine our evaluation of the pig model of maturity in the context of iron metabolism studies. Our results prove preterm piglets to be a promising and competitive model for the investigation of iron metabolism in premature infants.

## 4. Materials and Methods

### 4.1. Animals and Experimental Design

The experiment was carried out in the Laboratory of Large Animal Models (The Kielanowski Institute of Animal Physiology and Nutrition of the Polish Academy of Sciences, Jabłonna). In the experiment, we used 3 Polish Landrace sows. The animals, 35–40 days after insemination (confirmed by ultrasound examination), were transported to a pig farm in Jabłonna. The animals were housed in individual pens and fed a balanced diet twice daily according to the nutritional requirements of the animals and stage of gestation. The animals had constant access to water ad libitum. Naturally, abortion does not occur in pigs, and pharmacologically induced delivery can result in fetal death due to large litters. For this purpose, healthy cull sows were selected from the herd. Due to the age and number of litters, the sows would still be sold to the slaughterhouse after giving birth (reduced breeding value). Cesarean section was manipulated on the 109th day of gestation to obtain premature piglets. After cesarean sections, 24 healthy premature piglets of both sexes were obtained. Six premature piglets were weighed and sacrificed for sampling immediately after birth. As a control, 6 naturally born Polish Landrace piglets (an average gestation period of 115.4 days) from 3 sows and of both sexes were sampled immediately after birth. The rest of the premature piglets were housed in special cages equipped with dry bedding and heating pads and fed via an intragastric tube for several consecutive days until they began to take food from a bottle with a teat. These piglets were used to observe postnatal growth and physiology.

### 4.2. Biological Sample Collection

Blood was collected immediately after full-term and preterm birth directly from the heart (under isoflurane anesthesia, 1.28%) at slaughter into tubes with heparin. Following an overdose of sodium pentobarbital by intravenous injection (100 mg/kg bw), liver and spleen samples were rapidly dissected and flushed with PBS. Samples of tissues were immediately dissected and fixed in a 4% formaldehyde solution (#1004968350, Sigma-Aldrich, St. Louis, MO, USA). The remaining portions were frozen in liquid nitrogen prior to storage at −80 °C until used for analysis. Bone marrow cells for Perls’ staining were rinsed from femurs with ice-cold Hanks’ balanced salt solution, then smeared onto slides and fixed with methanol.

### 4.3. Measurement of RBC Indices and Blood Plasma Iron Parameters

RBC indices were determined using IDEXX ProCyte Dx, an automated hematology analyzer (IDEXX Laboratories, Westbrook, ME, USA). The colorimetric measurement of an iron–chromazurol complex (absorbance at 630 nm) was used to measure iron concentration in the blood plasma (#1-420-0200, Biomaxima, Lublin, Poland) and TIBC (#1-421-0060, Biomaxima, Lublin, Poland). The percentage of TSAT was then calculated according to the following formula: TSAT = (plasma iron/TIBC) × 100. The plasma ferritin concentration was evaluated by a porcine ELISA kit (#EP0255, Fine Biotech, Wuhan, China).

### 4.4. Measurement of Iron Content in Tissues

The tissular non-heme iron content was determined by acid digestion of the samples at 100 °C for 10 min, followed by colorimetric measurement of an iron–ferrozine complex (absorbance at 562 nm, Beckman DU-68, Beckman Coulter, Brea, CA, USA) [48].

### 4.5. Perls’ Staining

After a 24 h fixation, segments of the liver and spleen were dehydrated, embedded in paraffin, and cut into 5 μm sections with a Hyrax M25 rotary microtome (Zeiss, Oberkochen, Germany). After mounting on glass slides, sections were deparaffinized, stained with Perls’ Prussian blue for 30 min, counterstained with nuclear red (#1001210500, Sigma-Aldrich, St. Louis, MO, USA) for 2 min and analyzed under a light microscope (Eclipse E200, Nikon, Amsterdam, The Netherlands). The same procedure of staining with Prussian Blue was performed with bone marrow smears prepared from piglets.

### 4.6. Measurement of Plasma Hepcidin, Erythropoietin, Erythroferrone, and GDF15 Level

For quantification of plasma hepcidin, erythropoietin, erythroferrone and GDF15 level, we used commercial ELISA kits (human Hepcidin 25, #EIA-5782, DRG Instruments GmbH, Marburg, Germany; porcine erythropoietin, #ABIN6955643, Antibodies-online, Aachen, Germany; porcine erythroferrone, #E07E0518, BlueGene Biotech, Shanghai, China; and porcine GDF15, #E07G0127, BlueGene Biotech, Shanghai, China) according to the manufacturer’s guidelines. The human Hepcidin 25 ELISA kit was validated for use in pigs in our previous study [49].

### 4.7. RNA Isolation and Real-Time Quantitative RT-PCR Analysis

Total RNA was isolated from about 30 mg of wet tissue using the SV Total RNA Isolation System (#Z3105, Promega, Madison, WI, USA). mRNA levels were measured by a real-time quantitative RT-PCR of cDNA derived from specific transcripts in the LightCycler^®^ 96 Instrument (Roche Diagnostics, Basel, Switzerland), using the pair of primers shown in Table S1. The amplified products were detected using SYBR Green I (#06924204001, Roche Diagnostics, Basel, Switzerland) according to the manufacturer’s guidelines. To confirm amplification specificity, the PCR products were subjected to melting curve analysis and agarose gel electrophoresis. Transcript levels were normalized relative to those of control reference genes selected using NormFinder software (MOMA, Aarhus, Denmark) (https://www.moma.dk/software/normfinder (accessed on 13 February 2023)). The expression of target genes versus housekeeping genes (GAPDH or HPRT) was calculated by the formula 2^−ΔΔCT^ [50].

### 4.8. Western Blot Analysis

To determine protein levels of L-ferritin, H-ferritin and ferroportin, 40 µg of membrane and cytosolic extracts were prepared and separated by electrophoresis on either 9% or 16% SDS-PAGE gels depending on the molecular weight of the protein [51]. Electroblotting of the resolved proteins onto the PVDF transfer membrane (#88518, Thermos Scientific, Waltham, MA, USA), blocking, and incubation with primary and secondary antibodies were performed according to previously described methods [51]. Table S2 shows the details of the primary and secondary antibodies. For quantitative analysis of protein content, reactive bands were quantified relative to actin using a Molecular Imager with Quantity One 4.6 software (Bio-Rad, Hercules, CA, USA).

### 4.9. Statistical Analysis

The results were statistically analyzed using independent samples t-tests using SPSS Statistics 26 (IBM, Armonk, NY, USA). Figures were prepared using GraphPad Prism 9.1.1 (GraphPad Software, San Diego, CA, USA). *p* < 0.05 was considered significant. Data are presented as mean values ± SEM.

## Data Availability

Data will be available upon request.

## Supplementary Materials

The following supporting information can be downloaded at: https://www.mdpi.com/article/10.3390/ijms252011215/s1.
