# Supplementary material for: Preterm Piglets Born by Cesarean Section as a Suitable Animal Model for the Study of Iron Metabolism in Premature Infants
Source: Int J Mol Sci. 2024 Oct 18;25(20):11215. doi: 10.3390/ijms252011215 (PMC11508764; doi:10.3390/ijms252011215)
Supplement: Supplementary file 1 [file ijms-25-11215-s001.zip › ijms-3235066-supplementary.pdf]

Table S1. Specific primer sequences for pigs used for real-time PCR

Gene	Forward (5' - 3')	Reverse (5' - 3')	Amplicon Size, bp	Gene Bank
Bone morphogenetic protein 6	TTGTGAACCTGGTGGAGTAC	GATTCGGAATTCTGCAGCCG	122	NM_001168001.1
Ferroportin	GCAGCAAAAGAACGAGTGGG	AAGGATCCACAGCATCCTCC	84	XM_003483701.4
GAPDH	CCCCTTCATTGACCTCCACT	CCCATTTGATTTTGGCGGGA	158	AF017079.1
Hepcidin	ATCCCAGACAAGACAGCTCA	TCTTGCAGCACATCCCACAGA	164	NM_214117.1
HPRT	GGCCATCACATCGTAGCCCT	TCGCCCGTTGACTGGTCATT	164	NM_001032376.2
Interleukin-6	AAGGTGATGCCACCTCAGAC	TCTGCCAGTACCTCCTTGCT	151	JQ839263.1
Tumor necrosis factor α	AAGACACCATGAGCACTGAGA	CGACCAGGAGGAAGGAGAAG	132	JF831365.1

Table S2. Antibodies used for western blot analysis

Target protein	Primary Ab	Dilution	Secondary Ab	Dilution
Ferroportin	Rabbit polyclonal, Alpha Diagnostic, #MTP11-A	1:1000	Goat anti-rabbit polyclonal, Sigma-Aldrich, #A6154	1:10000
β-actin	Mouse polyclonal, Thermo Fisher Scientific, #MA5-11869	1:1000	Goat anti-mouse polyclonal, Sigma-Aldrich, #A5278	1:10000
Ferritin light chain	Rabbit polyclonal, ABCAM, #ab69090	1:1000	Goat anti-rabbit polyclonal, Sigma-Aldrich, #A6154	1:10000
Ferritin heavy chain	Rabbit polyclonal, ABCAM, #ab65080	1:1000	Goat anti-rabbit polyclonal, Sigma-Aldrich, #A6154	1:10000

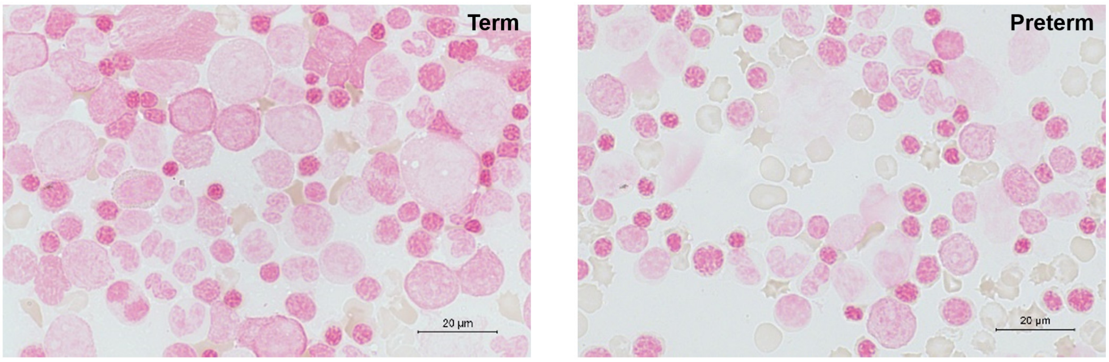

Figure S1. Histological examination of iron loading in bone marrow of piglets. Original magnification: 1000×. Scale bars = 20µm.

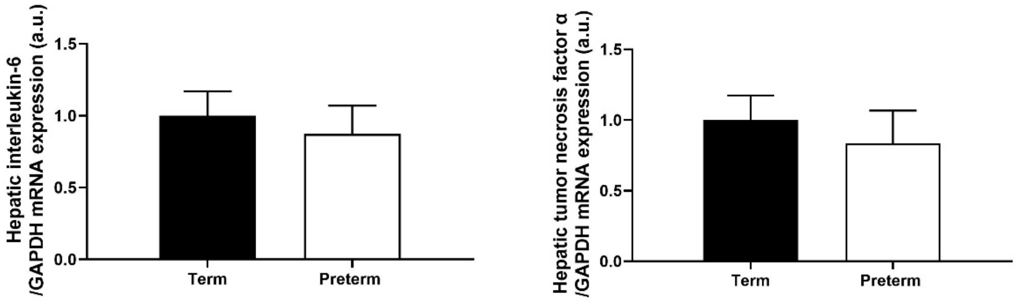

Figure S2. Inflammatory cytokines mRNA expression in the liver. Data are presented as the mean ± SEM (n = 6). a.u., arbitrary units.
